# Fine-Grained Age-Class Identification of Moso Bamboo Using an Improved Lightweight YOLO11 Model

**DOI:** 10.3390/jimaging12030102

**Published:** 2026-02-27

**Authors:** Yingbin Zhang, Xinhuang Zhang, Zhichao Cai, Xi He, Shuwei Chen, Zhengxuan Lai, Kunyong Yu, Riwen Lai

**Affiliations:** College of Forestry, Fujian Agriculture and Forestry University, No. 15 Shangxiadian Road, Fuzhou 350002, China; fafuzyb@fafu.edu.cn (Y.Z.); fafuzxh@fafu.edu.cn (X.Z.); fafuczc@fafu.edu.cn (Z.C.); fafuhexi@fafu.edu.cn (X.H.); fafucsw@fafu.edu.cn (S.C.); fjlzxfjlzx@163.com (Z.L.); yuyky@126.com (K.Y.)

**Keywords:** moso bamboo, culm age classification, YOLO11, lightweight deep learning, fine-grained recognition

## Abstract

Accurate identification of moso bamboo (*Phyllostachys edulis*) age classes is essential for effective forestry resource management, yet existing methods often struggle to achieve a satisfactory balance between accuracy and computational efficiency under complex field conditions. To address this challenge, this study proposes a lightweight object detection model, termed YOLO11-GCR, for fine-grained moso bamboo age-class classification based on close-range imagery. The proposed approach builds upon the YOLO11 framework and incorporates Ghost convolution, the Convolutional Block Attention Module (CBAM), and a Receptive Field Block (RFB) to reduce model complexity, enhance discriminative feature representation, and improve sensitivity to subtle texture variations among age classes. A dataset consisting of 9538 annotated bamboo culm images covering four age classes (I-du to IV-du) was constructed and divided into training, validation, and independent test sets with strict spatiotemporal separation. Experimental results indicate that YOLO11-GCR achieves robust detection performance with a lightweight architecture of 2.62 × 10^6^ parameters and 6.2 GFLOPs, yielding an mAP@0.5 of 0.913 and an mAP@0.5–0.95 of 0.895 on the independent test set. Notably, the model demonstrates improved classification stability for visually similar age classes, such as II-du and III-du. Overall, this study presents an efficient and practical imaging-based solution for automated moso bamboo age-class recognition in complex natural environments.

## 1. Introduction

Moso bamboo (*Phyllostachys edulis*) is the most widely distributed bamboo species in China and possesses outstanding economic and ecological value. Its age-class structure directly determines bamboo forest management strategies, harvesting cycles, and carbon sequestration capacity [1,2]. In practical bamboo forest management, the growth stages of moso bamboo are commonly classified according to a chronological age-based system, with “du” used as the unit of age class. Specifically, newly emerged bamboo shoots that complete rapid growth within the current year are defined as I-du; bamboo aged 2–3 years is classified as II-du; bamboo aged 4–5 years is categorized as III-du; thereafter, the age class increases sequentially with approximately every additional two years of growth [3,4]. This age-classification scheme comprehensively reflects physiological characteristics and material property differences across different growth stages of moso bamboo [5,6] and has been widely adopted as an empirical standard in bamboo harvesting decisions, yield estimation, and resource management [7].

At present, however, moso bamboo age determination still relies predominantly on manual experience, typically through visual inspection of phenotypic traits such as culm color, internode morphology, and surface powder characteristics [8]. Influenced by seasonal variation, illumination conditions, and the complexity of understory environments, these phenotypic features tend to exhibit instability across different habitats. This not only increases subjectivity in age-class determination but also limits its applicability and efficiency in large-scale bamboo forest management [9]. Although previous studies have demonstrated that information such as laser scanning echo intensity can partially assist bamboo age identification [3], the high cost of related equipment and the restrictive data acquisition conditions [10] hinder their widespread use in routine bamboo forest surveys and management practices.

In recent years, deep learning techniques have shown remarkable advantages in fields such as plant phenotypic recognition, tree crown detection, and crop health monitoring [11,12,13,14,15]. Image-based acquisition combined with deep learning algorithms for automatic detection and classification has become increasingly more advanced [16,17,18]. Recent studies have further demonstrated that task-oriented improvements of YOLO-based architectures can significantly enhance fine-grained plant detection performance under complex natural backgrounds [19], highlighting the effectiveness of integrating architectural refinement and feature enhancement strategies for subtle morphological discrimination. Nevertheless, although mainstream object detection algorithms such as YOLO [20] and Faster R-CNN [21] have achieved excellent performance on standard image datasets, their direct application to moso bamboo age recognition still faces multiple challenges. On the one hand, differences in texture, coloration, and epidermal structure among different du age classes are subtle, and adjacent classes (e.g., II-du and III-du) exhibit continuous transitional characteristics. This constitutes a typical fine-grained visual recognition problem [22], in which generic object detection frameworks are prone to confusion when handling weakly discriminative features. Similar challenges have been reported in intelligent monitoring systems for ornamental and horticultural crops, where enhanced YOLO-based detectors were employed to distinguish subtle growth-stage variations in visually homogeneous plant structures [23], further emphasizing the necessity of task-specific architectural adaptation in fine-grained plant recognition tasks. On the other hand, complex illumination conditions, partial occlusion, and background color similarity commonly present in bamboo forest environments further weaken the model’s responsiveness to age-discriminative features [24]. In addition, there is still a lack of moso bamboo image datasets that cover multiple regions and age classes with reliable label sources [25], which constrains the development and validation of task-specific models to some extent.

In recent years, lightweight object detection architectures have attracted increasing attention, particularly in real-world deployment scenarios where computational efficiency and inference speed are critical. Although high-capacity models often achieve superior benchmark performance, their substantial parameter counts and computational overhead may limit practical applicability in field environments. Consequently, numerous studies have explored structural optimization strategies to achieve a better balance between representational capability and computational cost. Representative approaches include efficient convolutional designs and feature redundancy reduction mechanisms, such as GhostNet [26], which generates more feature maps from inexpensive operations, as well as lightweight backbone architectures exemplified by MobileNetV3 [27] and ShuffleNetV2 [28] that provide practical guidelines for efficient network design. Moreover, deployment-oriented detection frameworks developed in safety-critical domains, such as RailVoxelDet [29] for railway LiDAR perception, further demonstrate that architectural simplification, efficient feature encoding, and attention-enhanced modules can effectively maintain detection precision under strict computational constraints. Collectively, these studies highlight a broader trend in modern object detection research toward structural efficiency and balanced performance optimization. To address these challenges, this study proposes a lightweight object detection model, termed YOLO11-GCR, specifically designed for moso bamboo culm detection and age-class classification. Based on the YOLO11 architecture, GhostConv is introduced to reduce redundant convolutional computations and enhance lightweight feature representation; a channel–spatial attention mechanism (CBAM) is incorporated to emphasize texture details highly relevant to age discrimination while suppressing understory background interference [15]; meanwhile, a multi-scale receptive field block (RFB) is embedded into the neck network to strengthen the model’s capability to capture texture features at different scales. In addition, a dataset comprising 9538 close-range field images of moso bamboo culms, covering age classes from I-du to IV-du, was constructed to provide reliable support for model training and performance evaluation [5,30].

The main contributions of this study are as follows: (1) A close-range moso bamboo forest image dataset covering multiple representative production regions was constructed. The age-class labels were derived from long-term plot monitoring data and generated through a rigorous expert-driven annotation framework, incorporating independent dual-annotator labeling, arbitration by a senior expert, and systematic quality control, thereby ensuring high objectivity, consistency, and reliability of the annotations. (2) A lightweight moso bamboo age detection model, YOLO11-GCR, was proposed, in which the synergistic design of GhostConv, CBAM, and RFB enhances the representation of fine-grained texture features. (3) Systematic comparative and ablation experiments were conducted to verify the model’s comprehensive advantages in detection accuracy and computational efficiency, particularly demonstrating more stable recognition performance for age classes with subtle texture differences, such as II-du and III-du. (4) Visualization-based analyses were employed to elucidate the mechanisms by which the improved modules influence feature attention regions, providing methodological references for the design of intelligent moso bamboo recognition models. (5) By optimizing the model to reduce computational costs, this study aims to enable near-real-time moso bamboo age recognition directly on mobile devices (e.g., smartphones). This design bridges the gap between algorithmic research and field investigations, allowing forestry practitioners to conduct on-site assessments without relying on cloud computing or bulky hardware.

In summary, from a practical forestry management perspective, this study aims to provide an efficient, lightweight, and robust technical solution for the automated determination of moso bamboo age classes under complex understory environments, thereby offering both data support and methodological guidance for intelligent bamboo forest management and ecological monitoring.

## 2. Materials and Methods

### 2.1. Study Area

**Figure 1:**
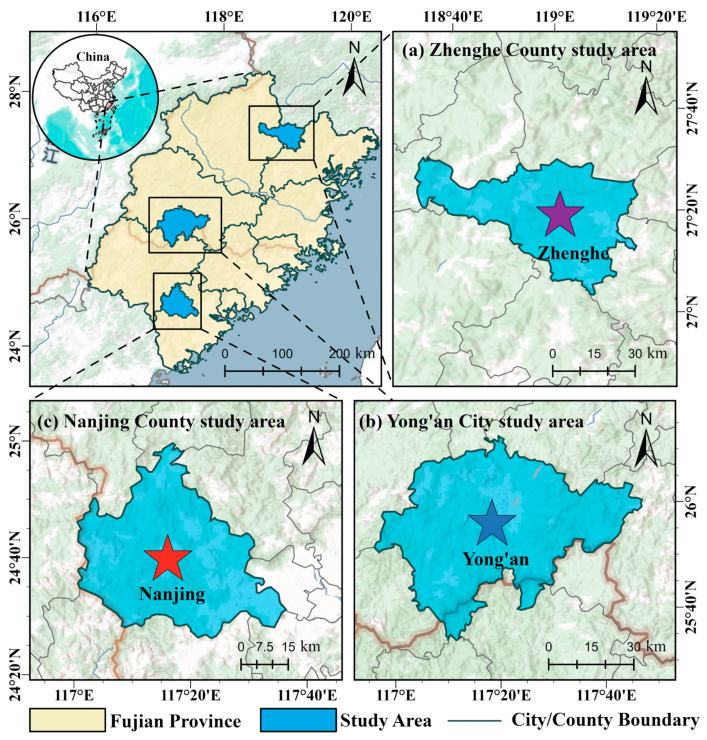
Overview of the study area. (**a**–**c**) Spatial distribution of the three study areas in Zhenghe County, Yong’an City and Nanjing County, Fujian Province.

Data collection for this study was conducted in three representative moso bamboo (*Phyllostachys edulis*) production regions in Fujian Province, China: Yong’an City, Zhenghe County, and Nanjing County (Figure 1). All study areas are located within the subtropical monsoon climate zone, characterized by distinct seasons and abundant precipitation, which provide favorable conditions for the growth and expansion of moso bamboo [31,32]. The regions exhibit a mean annual temperature of approximately 17–20 °C and an annual precipitation generally exceeding 1600 mm. Notable environmental features include pronounced variations in illumination, high atmospheric humidity, and complex understory conditions, collectively offering diverse habitat settings for the natural phenotypic differentiation of moso bamboo across different age classes [33,34].

The three sampling areas differ in topography, management practices, and stand structure, thereby representing typical site conditions of moso bamboo forests in Fujian Province [30,35]. Bamboo forests in Yong’an City are characterized by large, contiguous distributions and a long history of intensive management, resulting in relatively uniform stand structures. In contrast, Zhenghe County features more fragmented terrain with pronounced altitudinal variation, leading to greater heterogeneity in understory illumination and background conditions. Bamboo forests in Nanjing County are primarily managed in a more dispersed manner, with marked differences in stand age structure. These regional distinctions not only enhance the diversity of the collected samples but also contribute to improving the model’s capacity to learn age-related bamboo features under varying environmental conditions [34,36].

Overall, the representativeness of moso bamboo resources and the environmental heterogeneity across the three regions make them well suited for constructing a diverse age-class dataset. This diversity is conducive to enhancing the generalization performance of the proposed model across different stands and complex background conditions [30,31].

### 2.2. Dataset Construction

#### 2.2.1. Fieldwork Design

**Figure 2:**
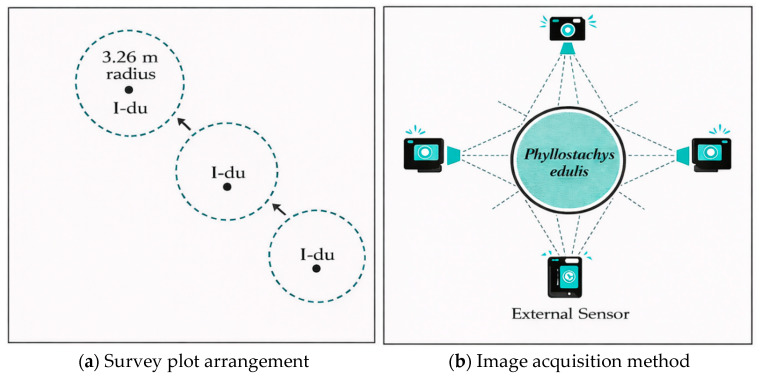
Survey plot arrangement and image acquisition method. (**a**) Circular plots centered on target bamboo culms (I-du). (**b**) Four-direction image acquisition around *Phyllostachys edulis*.

In each sampling region, three consecutive standard circular plots with a radius of 3.26 m were established in a linear arrangement, using newly emerged bamboo of the current year (I-du) as the central reference point, as shown in Figure 2a. All plots were designed as permanent sample plots to avoid spatial displacement caused by harvesting activities. Each moso bamboo culm within the plots was assigned a unique identifier, and its annual growth status was continuously recorded, ensuring the traceability and temporal stability of age-class labels [37]. To explicitly capture the seasonal variations in bamboo surface morphology influenced by changes in humidity, foliage density, and illumination conditions, fixed-point field observations were conducted during four distinct survey campaigns, spanning July 2024 (summer), August 2024 (summer), December 2024 (winter), and July 2025 (summer) [1,38].

**Figure 3:**
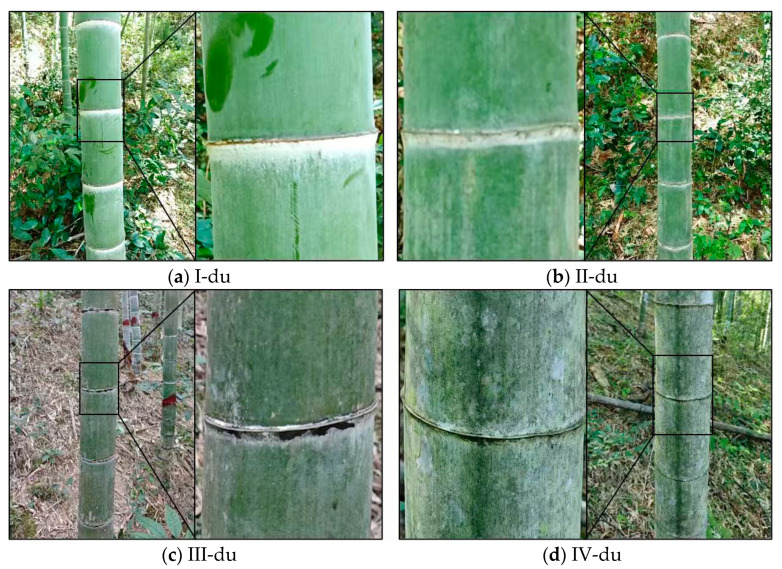
Representative examples of moso bamboo across different age classes. (**a**–**d**) highlight the age-related differences in culm color and surface texture among I-du, II-du, III-du, and IV-du bamboo, respectively.

Image acquisition was conducted using smartphone cameras, specifically current mainstream models including Apple iPhone 15, Huawei Pura 70, Xiaomi 15, Redmi K70, and Redmi K50. Although the smartphone models differ slightly in sensor specifications, all devices are equipped with high-resolution primary cameras, and images were captured in automatic mode to simulate typical shooting conditions in real-world applications. The middle-to-lower section of bamboo culms was selected as the primary imaging target, as this region contains the most discriminative age-related features, such as epidermal powder coverage, internode texture, and color variation [9,39]. To enhance the discernibility of texture details, the shooting distance was controlled within 1–1.5 m, and images of each bamboo culm were captured from four directions to account for directional differences in surface texture [40,41], as shown in Figure 2b. During image acquisition, occlusion and overexposure were minimized as much as possible, while natural variations in understory illumination and background conditions were intentionally preserved to improve the dataset’s representation of real-world environments [42]. Visual examples of bamboo culms across different age classes are shown in Figure 3.

#### 2.2.2. Preprocessing

To ensure consistency and quality of model inputs, the raw images were subjected to the following preprocessing steps: (1) A quality control procedure was applied to remove samples that were blurred, improperly exposed, or severely tilted. (2) All retained images were converted to JPEG format and resized to a resolution of 1080 × 1440 pixels. This resolution was selected based on preliminary experiments to balance the preservation of key texture details with the constraints of mobile inference while maintaining the original aspect ratio to avoid geometric distortion and reducing GPU memory consumption during training. (3) The AnyLabeling tool (Version 0.4.35) was used to annotate bounding boxes around bamboo culm regions and assign corresponding age-class labels [15].

#### 2.2.3. Ground-Truth Age-Based Annotation Workflow

To establish a reliable age annotation system, this study adopted a marker-based tracking strategy to construct verifiable ground-truth age labels. By attaching durable metal tags or applying long-lasting physical markings at the base of bamboo culms, the emergence year of individual bamboo was precisely recorded, enabling lifecycle-level traceability. Based on these physically marked individuals from three representative production regions, a stratified and balanced sampling strategy was employed to construct a benchmark dataset comprising 1200 images. Each image in this benchmark dataset corresponds to an unambiguous and verifiable true age uniquely determined by physical markers, serving as a “gold standard” for subsequent age annotation tasks.

**Figure 4:**
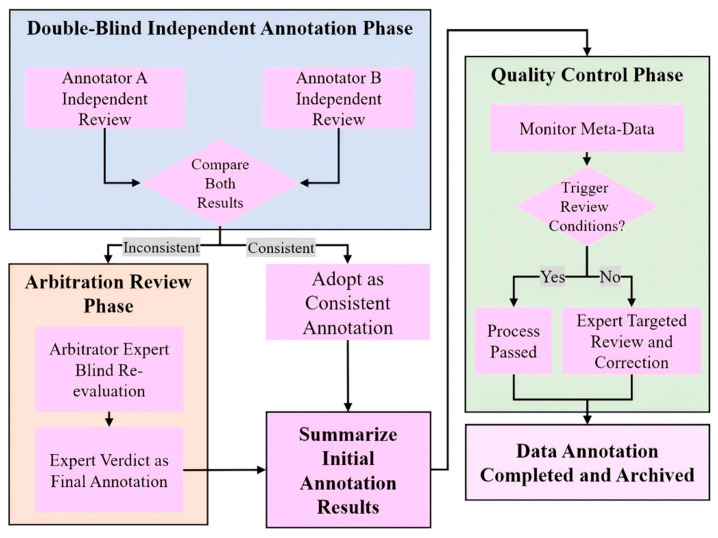
Workflow of the double-blind independent annotation and quality control process.

Building upon this benchmark dataset, a professional annotation team was assembled, consisting of two annotators with more than five years of moso bamboo field survey experience and one arbitration expert with over ten years of related expertise. All team members passed a specialized competency assessment based on the benchmark dataset, ensuring their ability to consistently identify key morphological traits across growth stages, including sheath ring characteristics, culm ring patterns, color, epidermal texture, and branching morphology. A standardized “dual independent annotation–expert arbitration–systematic quality control” workflow was then applied to annotate the remaining 8356 images without physical markers, and the overall workflow is illustrated in Figure 4. Specifically, two annotators independently performed age classification based solely on visual features, without access to each other’s results or external information. For samples with consistent annotations, labels were directly accepted; for the 1072 inconsistent samples (12.8%), blind re-evaluation was conducted by the arbitration expert, whose decision constituted the final label. In addition, system-level quality control automatically recorded annotation duration, revision frequency, and self-reported confidence to identify anomalous behaviors and trigger targeted rechecks. For high-risk borderline cases that remained uncertain after arbitration, focused re-evaluation was performed, resulting in the correction of 23 images (0.27% of the annotated samples), thereby ensuring high objectivity and consistency throughout the annotation process.

After preprocessing and multi-level quality control, the final dataset comprised 9538 images, including 1200 benchmark samples and 8356 annotated samples. An independent third-party stratified random validation (*n* = 500) indicated an overall age-labeling accuracy of 98.6% (493/500). The entire annotation process was governed by three layers of quality assurance: inter-annotator agreement measured by Cohen’s Kappa coefficient (0.87, indicating almost perfect agreement [13,24]), complete arbitration of all inconsistent samples, and continuous system-level quality control. Collectively, these measures ensure that the overall labeling accuracy of the dataset exceeds 95%, providing strong guarantees of scientific rigor, reliability, and generalizability.

#### 2.2.4. Dataset Splitting

**Figure 5:**
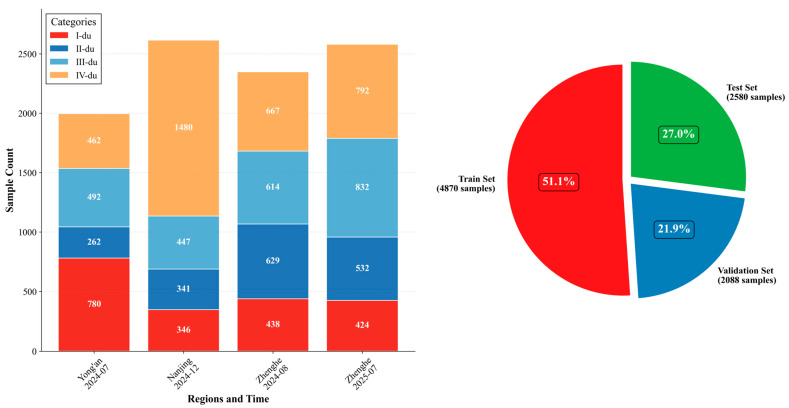
Distribution of moso bamboo image samples across age classes in different regions.

For model development and evaluation, the dataset was partitioned with explicit spatiotemporal independence. The training set comprised 4870 images (51.1%), randomly sampled from 70% of the 2024 data across three regions (Nanjing winter, Yong’an summer, and Zhenghe summer). The validation set contained 2088 images (21.9%), consisting of the remaining 30% of the 2024 data. The test set included 2580 images (27.0%), drawn exclusively from the independent 2025 summer survey in Zhenghe. To avoid information leakage, all images from the same location and time period were strictly assigned to the same subset. Unless otherwise specified, the comparative results are reported on the independent test set, while training curves and the ablation analyses are monitored on the validation set. The overall age-class distribution was as follows: I-du, 1988 images (20.8%); II-du, 1764 images (18.5%); III-du, 2385 images (25.0%); and IV-du, 3401 images (35.7%). The distribution of image samples across regions, acquisition periods, and age classes is illustrated in Figure 5. All comparative experiments were conducted using these fixed training, validation, and test sets to ensure fair and consistent evaluation.

Although semantic segmentation could provide pixel-level culm delineation, our primary goal is efficient age classification in complex natural backgrounds. To achieve this, we formulate the task as object detection rather than segmentation, as bounding-box detection offers a practical trade-off: (i) annotation cost is significantly lower, (ii) the model remains lightweight and suitable for mobile deployment, and (iii) the detection framework naturally supports future extension to multi-instance scenes. This design choice is reflected in our dataset, where although the current images primarily consist of single-instance annotations to control intra-image variability, they were all acquired under natural field conditions with dense and complex backgrounds. Each image contains one annotated bamboo culm instance, and the model is trained to distinguish the target from substantial background visual noise. This single-instance detection setting thus serves as a deliberate stepping stone toward future extension to dense multi-instance detection scenarios in complex bamboo forest environments.

### 2.3. Data Augmentation

To enhance the robustness of the proposed model under conditions of uneven understory illumination, complex backgrounds, and variable shooting perspectives commonly encountered in natural bamboo forests, a multi-type data augmentation strategy was implemented during training using the Albumentations library [43,44]. The augmentation scheme primarily comprised three categories: illumination–color augmentation, geometric transformation, and sample-mixing augmentation. The overarching objective was to improve the model’s adaptability to environmental variability while preserving age-related phenotypic characteristics of moso bamboo [45,46].

(1) Illumination–Color Augmentation (HSV-based)

The coloration and epidermal powder features of moso bamboo culms are highly sensitive to lighting conditions, and understory scenes are prone to instability caused by shadows and specular reflections. To simulate such illumination variability, perturbations were applied independently to hue (H), saturation (S), and value (V) channels with magnitudes of 0.015, 0.7, and 0.4, respectively. This strategy enhances the model’s robustness to color variations induced by changing lighting conditions [47,48,49].

(2) Geometric Transformation Augmentation

During field image acquisition in bamboo forests, shooting angles, distances, and framing are difficult to standardize. To emulate positional and scale variations arising from real-world image capture, geometric transformations including translation (ratio of 0.1), scaling (ratio of 0.5), and horizontal flipping (probability of 0.5) were applied. These operations expand the model’s capacity to adapt to diverse spatial layouts [50,51].

(3) Sample-Mixing Augmentation (Mosaic Augmentation)

Mosaic augmentation, which combines four images into a single composite image, effectively enhances the learning of local target features while increasing background complexity in training samples. Given the strong background interference typically present in bamboo forest environments, Mosaic augmentation was applied with a probability of 1.0 in this study to maximize its contribution to texture feature extraction [52,53].

**Figure 6:**
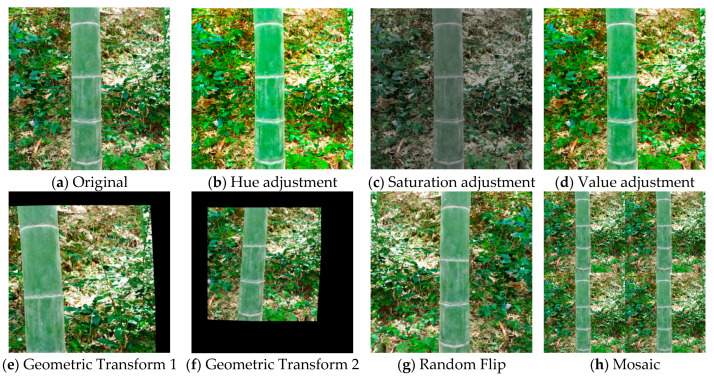
Illustration of data augmentation strategies. (**a**–**h**) show representative examples of different data augmentation methods.

Collectively, these augmentation operations constituted a diversified training data generation strategy, with representative augmented samples illustrated in Figure 6. By integrating multiple types of perturbations, the model is able to maintain higher feature stability and improved generalization performance in complex, real-world understory environments.

### 2.4. YOLO11-GCR Model Architecture

**Figure 7:**
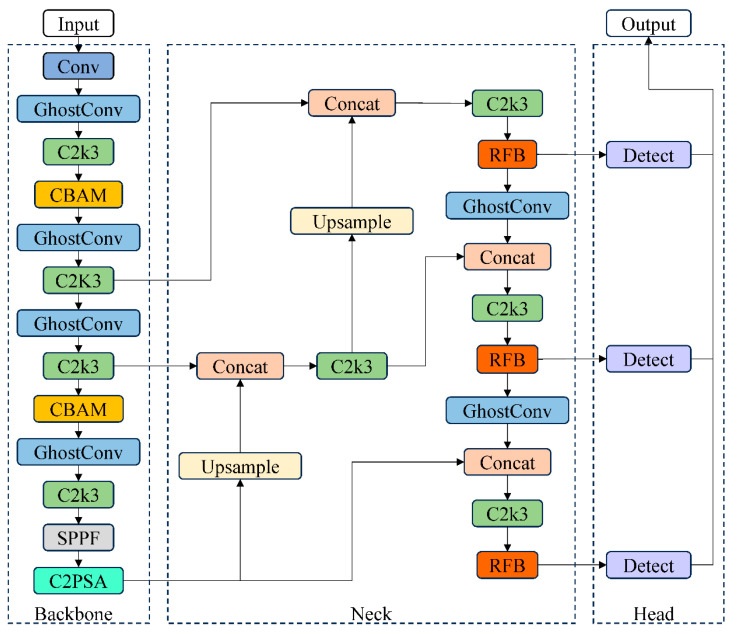
Architecture of the YOLO11-GCR.

YOLO11 is adopted as an advanced lightweight detection baseline derived from the YOLO family, serving as a representative modern single-stage detector for fine-grained object recognition tasks. The YOLO11-GCR model is developed based on the overall architecture of YOLO11 and consists of three main components: a backbone network, a feature fusion neck, and multi-scale detection heads [54]. The overall structure is illustrated in Figure 7. While preserving the high-efficiency detection characteristics of YOLO11, the proposed model introduces targeted improvements to the backbone, neck, and feature enhancement pathways to address the key challenges of moso bamboo age recognition, namely weak fine-grained texture cues, strong background interference, and pronounced scale variations. Specifically, lightweight convolutional modules are adopted in the backbone to improve feature extraction efficiency, attention mechanisms are incorporated at critical feature layers to strengthen responses to age-related textures, and a multi-scale receptive field structure is introduced in the neck to enhance the modeling of texture and structural features. Through these modifications, YOLO11-GCR maintains a compact architecture while significantly enhancing its ability to represent fine culm details, thereby providing more discriminative multi-scale feature representations for subsequent age classification.

#### 2.4.1. GhostConv-Based Lightweight Backbone

**Figure 8:**
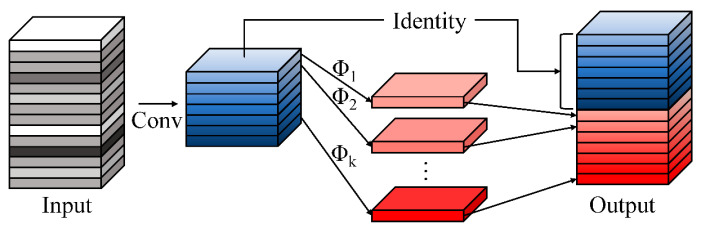
Architecture of GhostNet.

To improve model lightweightness and reduce redundant computations during feature extraction, YOLO11-GCR replaces the original convolutional structures in the backbone with GhostConv modules [55,56,57]. GhostConv is based on the principle of feature redundancy, where part of the feature maps is generated by standard convolutions, while the remaining features are efficiently approximated through inexpensive linear transformations. This design allows the network to substantially reduce parameter count and FLOPs while retaining sufficient feature representation capability [58]. The architectural principle of GhostNet is illustrated in Figure 8. As a result, GhostConv enables the model to achieve representational performance comparable to conventional convolutions at a significantly lower computational cost, thereby improving overall inference efficiency [59,60].

In the context of moso bamboo age recognition, culm texture differences are subtle and local feature distributions are relatively uniform; excessive convolutional redundancy may therefore impose unnecessary computational burdens. The introduction of GhostConv allows the backbone to extract texture and color features with reduced computational overhead, satisfying the representational requirements of fine-grained recognition while providing efficient feature inputs for subsequent attention mechanisms and multi-scale modules. Consequently, GhostConv establishes a lightweight, compact, and environment-adaptive backbone architecture for YOLO11-GCR that is well suited to complex understory conditions [61].

#### 2.4.2. CBAM Attention Module

**Figure 9:**
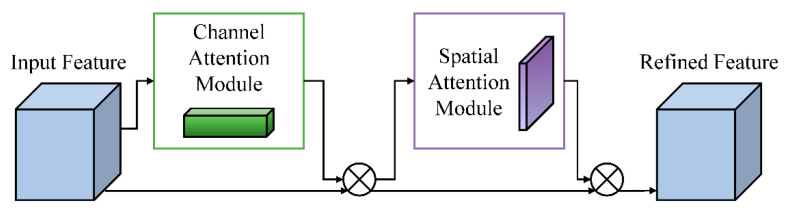
Structure of the CBAM module.

The discrimination of moso bamboo age classes primarily relies on subtle differences in surface texture and color, whereas understory environments often contain background interference such as leaves, branches, and mottled lighting, which can attenuate critical features during convolutional processing. To enhance the network’s focus on discriminative regions, YOLO11-GCR integrates the Convolutional Block Attention Module (CBAM) into key feature layers of the backbone [62,63]. CBAM sequentially models channel attention and spatial attention, enabling the network to simultaneously improve the selection of important feature channels and strengthen responses to key spatial regions [64,65]. The structure of the CBAM module is illustrated in Figure 9. This mechanism highlights age-relevant texture details while suppressing irrelevant background information during feature representation.

By incorporating CBAM into the lightweight backbone, the network achieves enhanced feature discriminability without incurring substantial additional computational cost. This module is particularly well suited to fine-grained texture recognition tasks in moso bamboo age classification, as it effectively alleviates feature confusion caused by weak inter-class texture differences and complex backgrounds. As a result, CBAM provides more stable and informative mid- to high-level semantic feature inputs for subsequent multi-scale feature fusion and age classification [66].

#### 2.4.3. RFB Multi-Scale Receptive Field Module

**Figure 10:**
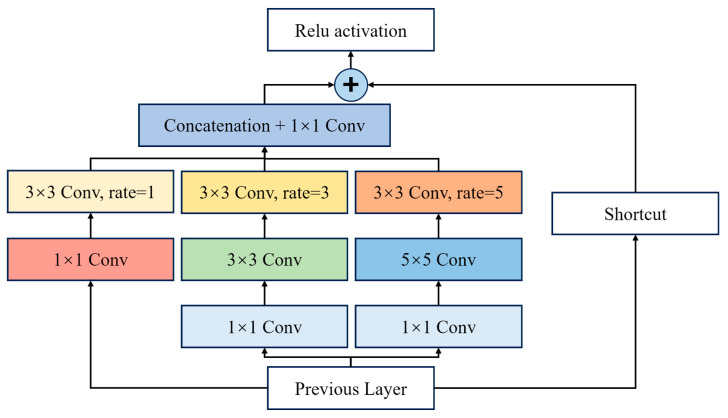
Structure of the RFB module.

Differences among moso bamboo age classes are often manifested in structural features with clear scale variability, such as internode thickness, texture granularity, and the spatial distribution of epidermal powder. Under close-range imaging conditions, variations in target size further exacerbate multi-scale disparities. To enhance the network’s sensitivity to such fine-grained structural variations, YOLO11-GCR introduces the Receptive Field Block (RFB) during the feature fusion stage [67]. RFB constructs effective receptive fields of different sizes through multi-branch dilated convolutions, mimicking the center–surround receptive field characteristics of the human visual system. This design strengthens the joint modeling of local textures and global structures [68,69], as illustrated in Figure 10. Consequently, the model’s ability to distinguish age-related differences in scale, texture density, and local structural patterns is significantly enhanced [70,71].

Within the overall architecture, RFB is embedded into the mid- to high-level feature fusion pathways of the neck to perform multi-scale enhancement of semantic features output from the backbone. This compensates for the limited receptive field coverage associated with lightweight convolutions and complements the feature selectivity reinforced by CBAM. By incorporating RFB, YOLO11-GCR achieves richer structural feature representations without substantially increasing computational complexity, thereby providing more discriminative multi-scale inputs for subsequent classification and localization in the detection heads [72].

### 2.5. Experimental Environment and Training Settings

Model training in this study was conducted on a Windows operating system. The experimental hardware configuration included a 12th Gen Intel Core i5-12490F CPU, an NVIDIA RTX 3060 GPU with 12 GB of video memory, and 32 GB of system RAM. The software environment consisted of Python 3.11, CUDA 12.1, and the PyTorch 2.5.1 deep learning framework to ensure training stability and reproducibility. The detailed training configuration and hyperparameter settings are summarized in Table 1.

We employed cosine annealing with a 3-epoch warm-up phase, during which the learning rate linearly increased from 0.001 to 0.01, followed by a cosine decay back to 0.001. To enhance training efficiency, we implemented an early stopping mechanism with a patience of 50, monitoring the comprehensive Fitness metric on the validation set. This Fitness score is defined as a weighted average of two key indicators: mAP@0.5 (weight: 0.1) and mAP@0.5–0.95 (weight: 0.9). In practice, training typically converged between 170 and 200 epochs, terminating before reaching the maximum limit, with an average stopping point at epoch 197 across three independent experimental runs.

Given that the proportion of IV-du samples was relatively high (35.7%), class weights were introduced into the classification loss to mitigate potential bias caused by class imbalance. The overall loss function followed the default YOLO training paradigm, consisting of a weighted classification loss, a bounding box regression loss, and an IoU-based localization loss. To ensure a fair comparison, all baseline models were trained using exactly the same optimization strategy and hyperparameter settings, thereby focusing the performance evaluation on differences in model architecture rather than training configurations.

Specifically, the class weight $w_{c}$ for each age class *c* was automatically computed based on the number of training samples $N_{c}$ as:

(1) $$\begin{array}{c} w_{c}=\frac{N_{total}}{C\times N_{c}} \end{array}$$

where $N_{total}$ denotes the total number of training samples and *C* represents the number of age classes. To ensure numerical stability during optimization, the maximum value of all class weights was capped at 10.0.

Based on the training set distribution (I-du: 1016, II-du: 900, III-du: 1216, IV-du: 1738), the computed class weights were: I-du = 1.198, II-du = 1.353, III-du = 1.001, IV-du = 0.700. After capping at 10.0, the weights remained unchanged (Table 2).

All training runs were performed using the fixed random seeds 42, 123, and 456 for the ablation experiments to quantify run-to-run variability.

### 2.6. Experimental Design and Evaluation Metrics

To systematically evaluate the performance of YOLO11-GCR, a comprehensive experimental framework was designed from three perspectives: module effectiveness validation, comparative model evaluation, and visualization-based analysis. This framework aims to thoroughly assess the model’s accuracy, efficiency, and feature representation capability in the task of moso bamboo age recognition.

#### 2.6.1. Ablation Study Design

To clarify the individual contributions and synergistic effects of the proposed improvement modules, a series of ablation experiments were conducted based on the original YOLO11 model. GhostConv, CBAM, and RFB were sequentially substituted or combined to construct multiple model variants for systematic comparison [73,74,75]. Single-module replacement experiments were used to analyze the impact of each module on feature representation and detection performance, while multi-module combination experiments were designed to evaluate their integrated optimization effects [76]. This experimental design facilitates validation of the effectiveness of each component in enhancing fine-grained texture recognition and multi-scale feature modeling.

#### 2.6.2. Comparative Experiment Design

To verify the comprehensive advantages of YOLO11-GCR in balancing lightweight design and detection accuracy, several representative single-stage and two-stage object detection models were selected as benchmarks, including YOLOv5 [77], YOLOv8 [78], YOLOv9t [79], YOLOv10 [80], SSD [81], and RTDETR [82]. Under unified training configurations, these models were compared in terms of detection accuracy, parameter count, and computational complexity (GFLOPs) to ensure fairness and reliability of the evaluation [83,84].

#### 2.6.3. Visualization-Based Analysis

To further provide intuitive insights into the differences in feature attention during age-class discrimination, two visualization approaches were adopted: detection result visualization and Grad-CAM heatmaps [85]. The former was used to compare localization accuracy and classification consistency across different models, while the latter was employed to analyze the extent to which the proposed improvement modules focus on key texture regions. These analyses help reveal changes in attention distribution during feature extraction and elucidate the underlying mechanisms of performance improvement [86,87].

#### 2.6.4. Evaluation Metrics

To comprehensively assess the detection performance of the YOLO11-GCR model, both detection accuracy and computational efficiency were evaluated. In terms of accuracy, precision, recall, average precision (AP), mean average precision (mAP), and the harmonic mean of precision and recall (F1-score), they were adopted as evaluation metrics [88]. In terms of computational efficiency, model parameters and floating-point operations (FLOPs) were used to evaluate the computational cost and hardware requirements of the model.

Precision (P) is defined as the proportion of samples that are actually positive among all samples predicted as positive by the model. Precision reflects the model’s false positive behavior and is calculated as

(2) $$P=\frac{T_{P}}{T_{P}+F_{P}},$$

where T_P_ denotes the number of samples correctly predicted as positive by the model, and F_P_ denotes the number of samples incorrectly predicted as positive.

Recall (R) is defined as the proportion of samples correctly predicted as positive by the model among all samples that are actually positive. Recall reflects the model’s false negative behavior and is calculated as

(3) $$R=\frac{T_{P}}{T_{P}+F_{N}},$$

where F_N_ denotes the number of samples incorrectly predicted as negative by the model.

Average Precision (AP) is defined as the area under the precision–recall (PR) curve, where recall is plotted on the horizontal axis and precision on the vertical axis. In other words, AP corresponds to the integral of the PR curve and is calculated as

(4) $$\mathrm{AP}=\int_{0}^{1}P\left(R\right)\mathrm{dR},$$

Mean Average Precision (mAP) is defined as the mean of the Average Precision (AP) values calculated across all predicted classes. Specifically, mAP@0.5 refers to the Average Precision computed at an intersection over union (IoU) threshold of 0.5, where IoU is defined as the ratio of the area of overlap between the predicted bounding box and the ground-truth bounding box to the area of their union. In contrast, mAP@0.5–0.95 represents the mean Average Precision averaged over multiple IoU thresholds ranging from 0.5 to 0.95. The calculation formula is given as

(5) $$\mathrm{mAP}=\frac{1}{M}\sum_{k=1}^{M}{\mathrm{AP}}_{k},$$

where AP_k_ denotes the Average Precision of the k-th class, and M represents the total number of classes. When calculating mAP@0.5, the Average Precision is computed at an IoU threshold of 0.5; when calculating mAP@0.5–0.95, the Average Precision corresponds to the mean value averaged over IoU thresholds ranging from 0.5 to 0.95.

The F1-score is defined as the harmonic mean of precision and recall and is used to balance these two metrics to reflect the overall performance of the model. The F1-score ranges from 0 to 1, with values closer to 1 indicating better overall performance. The calculation formula is given as

(6) $$F_{1}=\frac{2\mathrm{PR}}{P+R},$$

As classification is the primary objective of this study, a confusion matrix was employed to separately derive the classification accuracy for each du age class as well as the overall classification accuracy, which were used as the evaluation criteria.

## 3. Results and Analysis

### 3.1. Comparison of Training Convergence Performance

**Figure 11:**
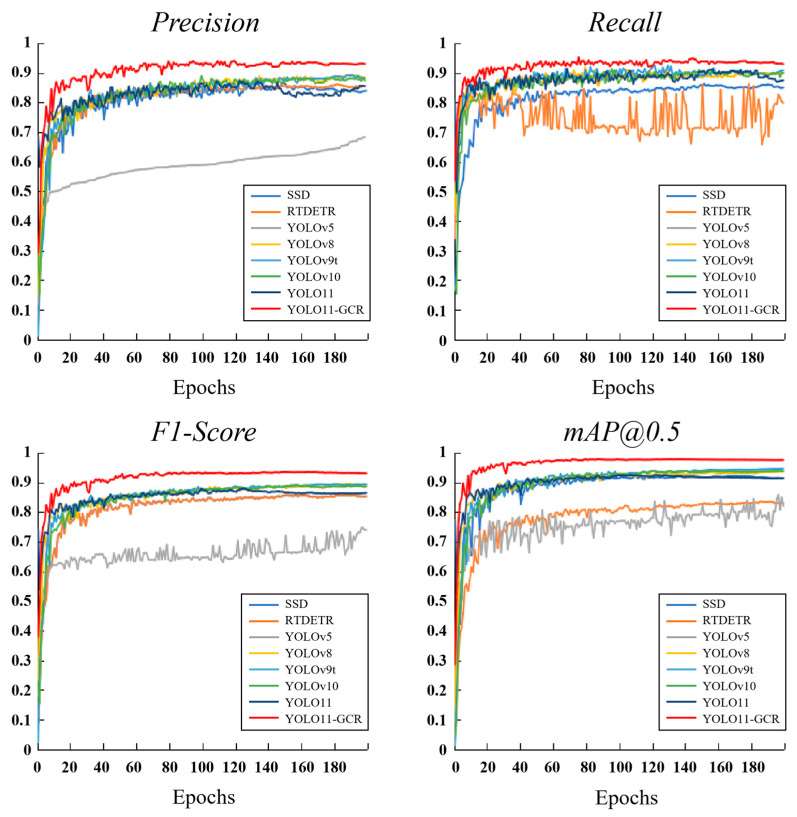
Training curves of key performance metrics. Training curves were monitored on the validation set under uniform training configurations and hyperparameters.

Figure 11 shows the training curves of four key metrics—Precision, Recall, F1-score, and mAP@0.5—across epochs for YOLO11-GCR and several baseline models. Under uniform training configurations and hyperparameters, YOLO11-GCR exhibits faster and more stable convergence. It achieves higher performance levels across all metrics at the end of training compared to the other models.

On the precision curves, YOLO11-GCR surpasses 0.90 within approximately the first 20 epochs and continues to improve steadily throughout training, ultimately reaching around 0.94. In contrast, YOLOv5 and YOLOv8 show relatively large fluctuations during the early training stage, with final precision values stabilizing at approximately 0.87. The consistently high precision achieved by YOLO11-GCR indicates a significantly lower false positive rate, enabling more reliable localization of bamboo culms, particularly in samples with complex backgrounds and subtle texture differences.

Regarding recall, YOLO11-GCR rapidly maintains a high level of around 0.92, whereas SSD remains below 0.85 for most of the training process, suggesting insufficient coverage of individuals across different age classes. RT-DETR exhibits a relatively slow convergence rate, which may be attributed to the limited adaptability of its feature fusion mechanism in fine-grained texture recognition scenarios. The superior recall performance of YOLO11-GCR reflects its stronger ability to detect targets with fewer missed detections.

In terms of the F1-score, which balances precision and recall, YOLO11-GCR consistently maintains values above 0.90 throughout training and ultimately reaches approximately 0.93, the highest among all evaluated models. Although YOLOv9t demonstrates relatively good performance, it still falls short of YOLO11-GCR, indicating that the proposed model achieves a more favorable balance between false positives and false negatives.

For the mAP@0.5 metric, which reflects overall detection accuracy, YOLO11-GCR attains a final value close to 0.98. This performance is notably higher than that of YOLOv8 (0.93), YOLOv10 (0.94), and YOLOv9t (0.95), and substantially superior to SSD (0.77) and YOLOv5 (0.80). It is worth noting that the baseline YOLO11 model achieves an mAP@0.5 of 0.974, which is further improved to 0.977 after integrating GhostConv, CBAM, and RFB. This improvement indicates that the synergistic multi-module design effectively enhances fine-grained feature modeling capability.

Taken together, the four training curves demonstrate that YOLO11-GCR converges smoothly throughout the entire training process with minimal oscillation, indicating more consistent gradient updates when handling fine-grained features such as bamboo culm texture and internode morphology. In contrast, fluctuations observed in the precision and recall curves of some comparative models suggest limited adaptability to weak-texture categories or partially occluded samples. These results confirm that the feature enhancement modules incorporated in YOLO11-GCR not only improve final detection performance but also significantly enhance training stability.

### 3.2. Module Effectiveness Analysis

Table 3 presents the performance variations achieved on the validation set by individually integrating GhostConv, CBAM, RFB, and their different combinations into the YOLO11 baseline model. The baseline model achieves an mAP@0.5 of 0.974 and an mAP@0.5–0.95 of 0.815 on the validation set, with 2.59 × 10^6^ parameters and 6.6 GFLOPs of computation, serving as the reference for subsequent ablation analyses. All experiments are conducted using three random seeds, and the results in the table are reported as mean ± standard deviation, with all metrics exhibiting low standard deviations.

(1) Individual Module Integration

We first examine the effect of introducing each module separately. Upon integrating GhostConv, the parameter count decreases from 2.59 × 10^6^ to 2.26 × 10^6^, and the computation reduces from 6.6 GFLOPs to 5.7 GFLOPs, representing reductions of approximately 14% and 13%, respectively. However, the mAP@0.5 slightly drops from 0.974 to 0.965 ± 0.003, indicating that while the lightweight operation effectively removes redundant features, it may introduce a certain degree of representation loss.

After adding CBAM, the mAP@0.5 improves to 0.968 ± 0.004, and the mAP@0.5–0.95 increases to 0.839 ± 0.006, demonstrating that the attention mechanism effectively enhances the network’s response to texture and spatial details.

When used individually, the RFB module shows the most significant performance gain in the high IoU threshold range, boosting the mAP@0.5–0.95 from 0.815 to 0.857 ± 0.003—an absolute gain of 0.042, which is the largest among all single-module configurations. Moreover, its mAP@0.5 (0.973 ± 0.001) remains highly stable, highlighting RFB’s effectiveness in capturing multi-scale texture features.

(2) Module Combination Effects

In the combination experiments, the components exhibit clear complementarity. The integration of GhostConv and CBAM restores the mAP@0.5 to 0.969 ± 0.005 and achieves an mAP@0.5–0.95 of 0.820 ± 0.007, while maintaining a low parameter count of 2.29 × 10^6^. Compared to using GhostConv alone, this represents a notable improvement, suggesting that CBAM can compensate for the feature loss caused by lightweight convolutions, although the stability of its mAP@0.5–0.95 requires further improvement.

The combination of GhostConv and RFB further enhances multi-scale feature extraction capability on the basis of a lightweight backbone. It achieves an mAP@0.5–0.95 of 0.842 ± 0.004, approaching the performance of RFB used alone, while keeping the computation within 6.8 GFLOPs and restoring the mAP@0.5 (0.974 ± 0.002) to the baseline level.

Although the combination of CBAM and RFB achieves a strong mAP@0.5–0.95 performance of 0.843 ± 0.005, the parameter count increases to 3.40 × 10^6^, a rise of approximately 30% compared to the baseline. This indicates a certain redundancy between the two modules, resulting in a less ideal trade-off between efficiency and performance compared to other combinations.

(3) Synergistic Integration of Three Modules and Summary

When GhostConv, CBAM, and RFB are synergistically integrated, YOLO11-GCR achieves the highest mAP@0.5 (0.977 ± 0.001) and an mAP@0.5–0.95 of 0.843 ± 0.003, while maintaining the parameter count at 2.62 × 10^6^ and reducing the computation to 6.2 GFLOPs. Compared to the baseline model, this represents a 0.028 improvement in mAP@0.5–0.95. This indicates that the synergistic integration of the three modules enhances both fine-grained feature representation and multi-scale modeling capability while preserving a lightweight architecture.

These results confirm the complementary roles of the three components in this task: GhostConv provides an efficient foundation for feature representation, CBAM strengthens the response to critical texture regions, and RFB enhances the discrimination of internode and texture variations across multiple scales, collectively constituting the optimal YOLO11-GCR architecture.

To further assess the generalization ability of YOLO11-GCR, Table 4 compares its performance on the validation set and the independent test set. The mAP@0.5 decreases slightly from 0.977 on the validation set to 0.913 on the test set, which is expected due to domain shift between the two datasets (e.g., different seasons and geographic locations). Notably, the mAP@0.5–0.95 increases from 0.843 to 0.895, indicating that the model achieves higher localization accuracy under stricter IoU thresholds on the test set. This improvement can be attributed to the test set being collected from a single survey campaign with more consistent imaging conditions (e.g., similar shooting distance and scale), which facilitates more precise bounding box regression. In contrast, the validation set contains greater variability in scale and appearance, making localization at high IoU thresholds more challenging. Overall, these results confirm that YOLO11-GCR generalizes well to new environments while maintaining robust localization performance.

Overall, the ablation experiments demonstrate that the proposed three-module collaborative design in YOLO11-GCR achieves the optimal balance between accuracy and efficiency. The stability of the results across multiple trials further validates the rationality and necessity of the proposed improvement strategy.

### 3.3. Overall Detection Performance Comparison

This subsection provides a global comparison of detection performance among different models on the test set to assess whether the proposed YOLO11-GCR offers a meaningful overall advantage. As summarized in Table 5, YOLO11-GCR achieves the highest mAP while maintaining a substantially lower parameter size and computational cost compared with heavier detectors. These results indicate that YOLO11-GCR provides a favorable balance between accuracy and efficiency under the same experimental setting. Unless otherwise specified, all evaluations in the following sections are performed on the test set.

Among the compared lightweight detectors (SSD, RT-DETR, YOLOv5, YOLOv8, YOLOv10, and the YOLO11 baseline), precision spans 0.785–0.877, recall 0.745–0.877, and F1-score 0.790–0.877. Their mAP@0.5 ranges from 0.740 to 0.899, and mAP@0.5–0.95 from 0.443 to 0.842. Parameter counts vary between 2.13 M and 32.97 M, while computational cost ranges from 6.6 to 33.9 GFLOPs. Notably, when evaluated using the efficiency metric mAP50 per GFLOPs, YOLO11-GCR achieves the highest value of 0.147, further underscoring its exceptional balance between detection accuracy and computational efficiency. None of these detectors simultaneously exceed YOLO11-GCR in all accuracy metrics, and most require substantially higher computational overhead.

YOLOv9t achieves a competitive mAP@0.5–0.95 of 0.854, yet this remains below the 0.895 attained by YOLO11-GCR. Moreover, YOLOv9t incurs 8.5 GFLOPs—27% higher than the 6.2 GFLOPs of YOLO11-GCR—while also exhibiting lower precision (0.905 vs. 0.919) and recall (0.895 vs. 0.922). Thus, its marginal performance does not justify the increased computational burden, reinforcing the efficiency advantage of the proposed model.

In summary, YOLO11-GCR achieves the highest values across key metrics such as Precision, Recall, F1-score, and mAP@0.5, while maintaining an optimal trade-off between mAP@0.5–0.95 and computational cost. This superior performance stems from its synergistic architectural design: the CBAM attention mechanism enhances focus on discriminative trunk textures, the RFB module captures multi-scale contextual features critical for size-variant age classes, and GhostConv ensures efficiency. Together, this design equips the model with robust feature representation for fine-grained discrimination in complex environments.

The following per-class analysis and visualizations will dissect how this enhanced capability specifically translates to performance gains on easily confused age categories and challenging real-world scenes.

### 3.4. Age-Class Classification Accuracy Across Different Models

**Figure 12:**
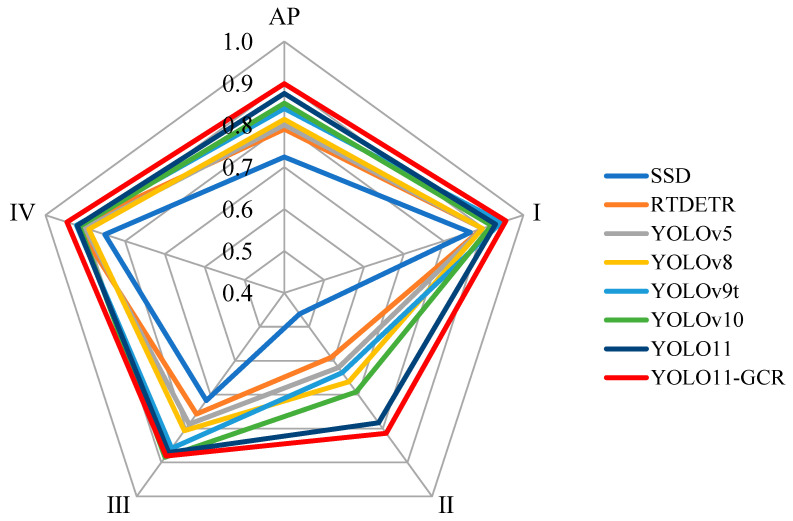
Radar chart of classification accuracy across different age classes. Each axis represents an age class (I-du, II-du, III-du, and IV-du).

**Figure 13:**
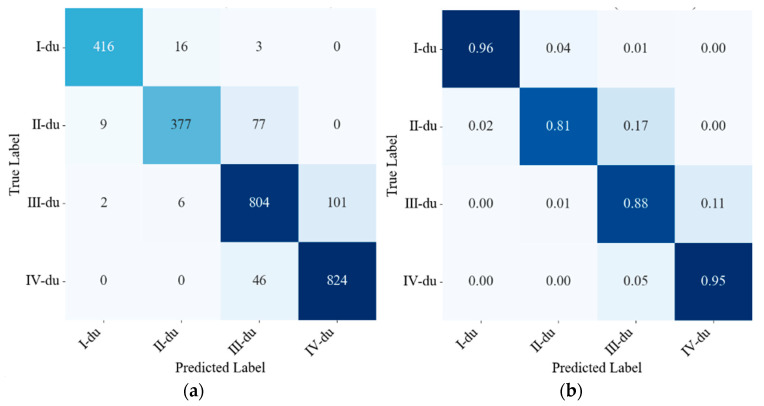
Confusion matrix. (**a**) Absolute Values; (**b**) Normalized. Rows represent the ground-truth labels, while columns denote the predicted labels. Highlighted values along the diagonal indicate the percentage of correctly classified samples, whereas off-diagonal elements represent misclassification. Darker colors correspond to higher counts or proportions.

YOLO11-GCR demonstrates the most balanced classification performance across all four age categories, particularly excelling in the visually subtle distinction between the II-du and III-du categories, thereby significantly reducing inter-category confusion. This section presents a fine-grained analysis of per-category classification performance through radar charts (Figure 12) and confusion matrices (Figure 13).

For class I-du, all evaluated models achieve high recognition accuracy, attributable to the distinctive phenotypic traits of young bamboo culms, such as brighter coloration and clearly visible powder coating. Nevertheless, YOLO11-GCR maintains the highest recognition accuracy in this category, reflected both by the longer radial length in the radar chart and the highly concentrated correct predictions in the diagonal region of its confusion matrix.

The most pronounced inter-model differences occur in classes II-du and III-du, which represent the most challenging age stages due to gradual and continuous texture progression. As shown in Figure 12, baseline detectors exhibit a clear performance drop along these two axes, suggesting frequent confusion between adjacent age categories. This pattern is further confirmed in the confusion matrix of YOLO11-GCR (Figure 13), where the majority of its off-diagonal errors are concentrated between II-du and III-du. Despite this intrinsic difficulty, YOLO11-GCR achieves diagonal classification accuracies exceeding 80% for both classes, indicating a substantial reduction in mutual misclassification compared with the baseline models.

For class III-du, although confusion with adjacent age stages remains observable, YOLO11-GCR shows a higher proportion of correct predictions than the baseline detectors, suggesting improved sensitivity to the subtle variations in texture and internode structure characteristic of this intermediate stage. This improvement is particularly significant because III-du often serves as a transition stage where phenotypic differences are visually subtle and easily affected by lighting and background interference.

In class IV-du, the classification performance of all models tends to converge, reflecting the more distinguishable rough texture and darker coloration of mature bamboo culms. Even under these conditions, YOLO11-GCR maintains a strong diagonal dominance in the confusion matrix, with the proportion of correct classifications exceeding 90%, demonstrating its robustness against cluttered backgrounds and age-related textural coarsening.

In summary, the key strength of YOLO11-GCR lies in its ability to reduce misclassification between adjacent and visually similar bamboo age classes, especially within the II-du–III-du transition zone. The model does not rely solely on easily distinguishable visual cues but exhibits enhanced discriminative power in the most error-prone age stages, thereby achieving a more balanced and reliable bamboo-age classification performance.

To further provide intuitive evidence of its detection robustness and classification rationale in real and complex understory environments, the following subsection presents qualitative visualizations and feature attention analyses.

### 3.5. Visualization Analysis

Qualitative visualization results indicate that YOLO11-GCR exhibits the most stable detection completeness, localization accuracy, and age-class consistency in complex understory scenes.

**Figure 14:**
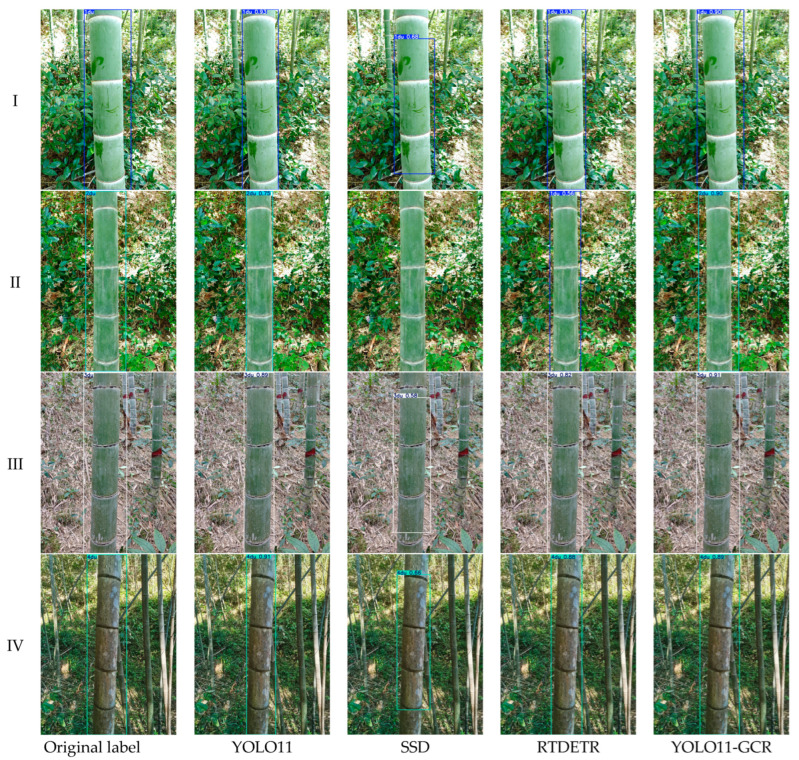
Detection results of different models across different age classes. Each row presents detection outputs from YOLO11-GCR, YOLO11, SSD, and RT-DETR for the same scene, including bounding boxes annotated with predicted age classes and confidence scores.

Figure 14 presents a comparative visualization of detection and classification results produced by YOLO11-GCR, YOLO11, SSD, and RT-DETR on moso bamboo samples across different age classes, covering typical scenarios such as complex understory backgrounds, uneven illumination, and partial occlusion. Overall, YOLO11-GCR demonstrates the most stable performance in terms of detection completeness, localization accuracy, and age-class consistency.

For I-du and II-du samples, both YOLO11 and YOLO11-GCR are able to correctly detect the targets; however, YOLO11-GCR exhibits superior bounding box alignment, particularly in regions where bamboo culms exhibit color similarity with the background. In such cases, the predicted bounding boxes of YOLO11-GCR are more compact and better fitted to the culm boundaries. In contrast, SSD shows missed detections in some II-du scenarios, mainly under low illumination or partial occlusion, indicating its limited sensitivity to low-contrast targets. Although RT-DETR is capable of detecting the targets, age-class misclassification occurs in certain samples, reflecting its limitations in fine-grained texture discrimination.

In III-du and IV-du scenarios, performance differences among models become more pronounced. YOLO11-GCR maintains accurate detection and stable classification even for these higher-difficulty age classes, whereas YOLO11 exhibits bounding box offsets or reduced confidence in some samples. SSD shows bounding boxes deviating from the culm body in III-du samples, suggesting insufficient adaptability to older bamboo features characterized by gradually coarsening textures and darkening coloration.

Overall analysis indicates that YOLO11-GCR exhibits stronger scene adaptability in complex understory environments, with its advantages mainly reflected in two aspects. First, the attention mechanism enhances the model’s focus on culm textures and internode structures, thereby reducing the impact of background interference on detection results. Second, the multi-scale receptive field structure enables accurate capture of variations in texture density and structural scale across different age classes. These characteristics allow YOLO11-GCR to achieve higher detection reliability in real-world applications, providing robust technical support for subsequent age classification and bamboo forest management.

### 3.6. Interpretability Analysis via Grad-CAM

**Figure 15:**
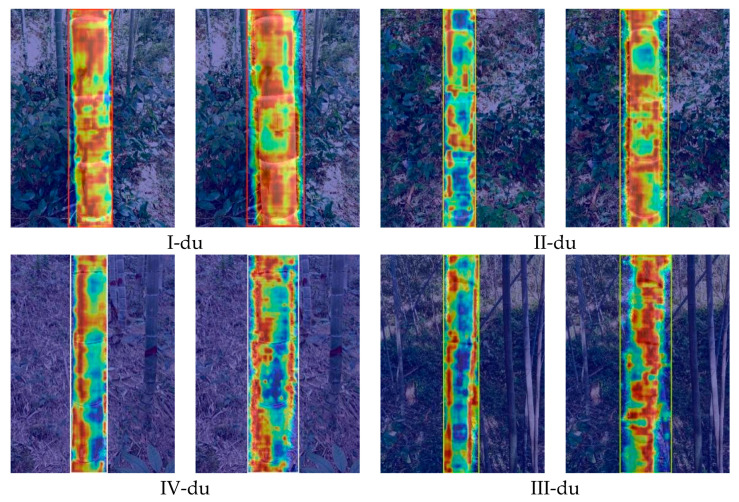
Comparative Grad-CAM heatmaps of the baseline model (**left**) and the improved model (**right**). The heatmaps are overlaid on the original input images. Warm colors (red and yellow) indicate regions with high attention during classification decisions, whereas cool colors (blue) denote regions with low attention.

To intuitively reveal the decision-making process and interpret the mechanisms behind the model’s classification performance, we employed Gradient-weighted Class Activation Mapping (Grad-CAM) to visualize the mid- to high-level feature responses of YOLO11-GCR and baseline models during age-class determination. The resulting heatmaps highlight the key image regions that contribute most to the classification decision, thereby allowing us to assess the rationality of feature extraction and the interpretability of each model (Figure 15).

Overall, the heatmaps of YOLO11-GCR are consistently focused on the bamboo culm body, especially on age-discriminative regions such as internode structures, epidermal textures, and powder distribution. In contrast, the baseline YOLO11 model often shows dispersed attention, with high-response areas extending into the understory background, adjacent foliage, or shaded regions, indicating weaker feature selectivity and higher susceptibility to environmental interference.

When examined by age class, all models produce relatively strong activations on the culm for distinct I-du samples; however, YOLO11-GCR yields more concentrated and boundary-sharp attention maps. The difference becomes most evident for the challenging II-du category, where inter-class differences are subtle. Here, baseline models frequently exhibit multiple scattered high-response zones, sometimes shifting focus away from core texture regions, reflecting instability in fine-grained discrimination. YOLO11-GCR, by comparison, maintains precise and stable attention on the most discriminative texture-variation areas in both II-du and III-du samples. For IV-du bamboo, whose coarser texture and darker color increase background interference, YOLO11-GCR still generates continuous and coherent activation over the culm, whereas the baseline model’s attention appears fragmented or diluted in some cases.

In summary, the Grad-CAM visualizations confirm that YOLO11-GCR adopts a more rational and task-aligned attention pattern. Its focused response on critical phenotypic regions directly explains the model’s improved accuracy and stability observed in the quantitative and qualitative results, especially for adjacent and visually similar age classes. This interpretability strengthens the reliability of YOLO11-GCR for practical deployment in complex understory environments.

### 3.7. Synthesis of Evidence

Synthesizing evidence from three aspects—quantitative metrics (Table 5), per-category AP analysis (Figure 11 and Figure 12), and visual detection results with attention maps (Figure 13 and Figure 14)—YOLO11-GCR not only outperforms comparison models numerically but also demonstrates distinct advantages in classification consistency, feature focus capability, and model interpretability within complex understory environments. This multi-perspective validation further supports its effectiveness and practicality for *Phyllostachys edulis* age identification, providing a solid foundation for subsequent lightweight deployment and field applications.

## 4. Discussion

This study addresses close-range moso bamboo age recognition, a fine-grained visual classification task, by introducing lightweight and feature-enhancement modifications to the YOLO11 architecture. Experimental results indicate that YOLO11-GCR achieves higher accuracy and computational efficiency than several baseline detectors. On the independent test set, the model reaches an mAP@0.5 of 0.913 and an mAP@0.5–0.95 of 0.895 with 2.62 × 10^6^ parameters and 6.2 GFLOPs, reflecting a favorable balance between precision and resource consumption.

Although each image in the dataset contains only a single bamboo culm, the task is formulated as object detection rather than pure classification. This design enables the model to first localize the culm region via bounding box regression, suppressing interference from complex backgrounds before fine-grained age classification. Moreover, the detection framework lays a foundation for future extension to multi-culm scenarios. From a methodological perspective, bamboo age determination depends on multiple subtle phenotypic cues—internode structure, epidermal texture, and powder distribution—that are spatially localized and scale-variant. By incorporating attention mechanisms and multi-scale receptive fields, YOLO11-GCR highlights age-relevant texture regions while capturing structural information across scales, reducing confusion among visually similar age classes such as II-du and III-du.

In terms of lightweight design, the results show that moderate reduction of redundant computations does not necessarily degrade performance, provided that discriminative features are preserved. GhostConv reduces parameter count and computational cost while offering an efficient feature representation foundation for subsequent attention and multi-scale modules. With 2.62 × 10^6^ parameters and 6.2 GFLOPs, the model maintains strong discriminative capability under low computational budgets—a key advantage for field surveys where rapid inference is needed on resource-limited devices.

To substantiate its practical efficiency, we measured inference speed on two platforms: an AMD Ryzen 5 4500U CPU (laptop) using the native PyTorch model (.pt) averaged 25.03 ms per image, while a Huawei Pura 70 smartphone using a TensorFlow Lite float32 model (CPU inference) required 128.15 ms per image. The sub-30 ms latency on the laptop suggests potential for real-time processing in portable field devices, and the 128 ms on a mobile phone remains acceptable for offline or on-demand analysis in bamboo forest surveys.

It is worth noting that II-du and III-du represent the most challenging categories, as their phenotypic characteristics exhibit gradual transitions with subtle texture differences sensitive to illumination and viewing angles. Per-class analysis shows that YOLO11-GCR consistently outperforms the baseline YOLO11 across all age classes, with particularly notable improvements for these visually similar categories. This improvement suggests that the attention mechanism’s focus on key texture regions, combined with multi-scale modeling of internode and texture variations, helps mitigate classification ambiguity arising from age continuity. This observation underscores the value of joint structure-texture modeling in fine-grained forest object recognition.

Despite the strong overall performance of YOLO11-GCR, several limitations remain. First, the dataset could be expanded to cover more geographic regions, seasonal conditions, and extreme weather scenarios. Second, the current age classes (I–IV du) are primarily visually defined; future work may explore more precise associations with physiological bamboo age. Third, although the model has been tested on representative hardware, its power consumption and performance on other edge platforms (e.g., UAVs) require further empirical validation.

Future research can be extended in several directions. First, recent advances in the DETR family, such as Roboflow RF-DETR—which performs well in detecting green objects under complex environments—may be integrated to further enhance performance. Second, ongoing developments in the YOLO series introduce architectural innovations that could benefit bamboo age recognition. For instance, YOLOv12 incorporates modules like C3k2_EMA and RFAConv that strengthen multi-scale feature extraction and fine-grained texture focus, potentially complementing the CBAM attention used in our model [89]. YOLOv13 offers hypergraph-enhanced perception and improved small-object detection [90], which may help locate partially occluded or distant bamboo culms. YOLO26 introduces end-to-end NMS-free inference and STAL, promising up to 43% CPU acceleration—directly relevant to our goal of lightweight field deployment [91]. YOLOE-26 further enables open-vocabulary recognition via a unified embedding space, allowing flexible age-class definition using natural language or reference images, which could reduce confusion between visually similar classes [92]. Although these models were released concurrently with or after our study, and their increased complexity would require careful adaptation to maintain deployment efficiency, future work could evaluate their applicability by integrating selective components (e.g., RFAConv, STAL) into the YOLO11-GCR framework or by retraining lightweight variants on our bamboo dataset. Finally, constructing a larger-scale multimodal bamboo forest dataset (e.g., incorporating near-infrared or hyperspectral imagery) and investigating vision transformer (ViT)-based feature extractors may further advance intelligent bamboo forest management.

## 5. Conclusions

This study addresses close-range moso bamboo age recognition by proposing a lightweight object detection model, YOLO11-GCR, and constructing an image dataset covering four age classes (I-du to IV-du). The dataset provides a foundation for model training and evaluation under field conditions.

Building upon the YOLO11 framework, the proposed approach introduces GhostConv, CBAM attention, and an RFB multi-scale module to enhance feature representation. Experimental results on an independent test set show that YOLO11-GCR achieves an mAP@0.5 of 0.913 and an mAP@0.5–0.95 of 0.895 with 2.62 × 10^6^ parameters and 6.2 GFLOPs. The model maintains higher accuracy than several baseline detectors while keeping computational cost low. Per-class analysis indicates that the largest improvements occur for the visually similar II-du and III-du categories, where classification is most challenging.

Ablation studies, detection visualizations, and Grad-CAM heatmaps further illustrate the contribution of each module. The lightweight backbone reduces parameter count while preserving feature representation capacity; the attention module strengthens responses to age-discriminative texture regions; and the multi-scale module captures structural variations across age classes. Together, these components improve both detection reliability and model interpretability in cluttered field environments.

Although YOLO11-GCR performs well on the current dataset, several limitations remain. Future work may expand the dataset to include more geographic regions and seasonal conditions, incorporate multi-view or temporal information to better capture continuous age-related changes, and explore integration with newer architectures such as YOLOv12, YOLO26, or YOLOE-26. These directions could further support the development of practical tools for bamboo forest management and resource inventory.

## Figures and Tables

**Table 1:** Training configuration and hyperparameter settings.

Parameter Category	Configuration
Input image size	640 × 640
Optimizer	Stochastic Gradient Descent (SGD)
Number of training epochs	200
Initial learning rate	0.01
Momentum	0.937
Weight decay	0.0005
Batch size	16
Learning rate schedule	Cosine annealing
Training strategy	Early stopping
Class imbalance handling	Class weights applied to the classification loss

**Table 2:** Class distribution and computed weights.

Class	Training Samples	Weight (Computed)	Weight (Capped)
I-du	1016	1.198	1.20
II-du	900	1.353	1.35
III-du	1216	1.001	1.00
IV-du	1738	0.700	0.70

**Table 3:** Ablation study on YOLO11 modules: Comparison of GhostConv, CBAM, and RFB. Evaluated on the validation set, the data are presented as mean ± standard deviation over three independent training runs with different random seeds. The best results are highlighted in bold.

YOLO11	GhostConv	CBAM	RFB	Params	GFLOPs	mAP@0.5	mAP@0.5–0.95
√				2.59 × 10^6^	6.6	0.974 ± 0.002	0.815 ± 0.005
√	√			2.26 × 10^6^	5.7	0.965 ± 0.003	0.839 ± 0.004
√		√		2.61 × 10^6^	6.5	0.968 ± 0.004	0.839 ± 0.006
√			√	2.68 × 10^6^	7.3	0.973 ± 0.001	**0.857 ± 0.003**
√	√	√		2.29 × 10^6^	5.7	0.969 ± 0.005	0.820 ± 0.007
√	√		√	2.39 × 10^6^	6.8	0.974 ± 0.002	0.842 ± 0.004
√		√	√	3.40 × 10^6^	9.9	0.972 ± 0.003	0.843 ± 0.005
√	√	√	√	2.62 × 10^6^	6.2	**0.977 ± 0.001**	0.843 ± 0.003

**Table 5:** Performance comparison of different object detection models on the independent test set.

Model	Params	GFLOPs	Prec.	Rec.	F1	mAP@0.5	mAP@0.5–0.95	mAP@0.5/GFLOPs
SSD	32.97 × 10^6^	33.9	0.785	0.796	0.790	0.740	0.443	0.022
RTDETR	7.71 × 10^6^	12.6	0.843	0.854	0.848	0.815	0.600	0.065
YOLOv5	2.51 × 10^6^	7.2	0.864	0.745	0.800	0.816	0.492	0.113
YOLOv8	3.16 × 10^6^	8.9	0.867	0.761	0.811	0.828	0.514	0.093
YOLOv9t	2.13 × 10^6^	8.5	0.861	0.895	0.878	0.905	0.854	0.106
YOLOv10	2.71 × 10^6^	8.4	0.877	0.783	0.827	0.845	0.582	0.101
YOLO11	2.59 × 10^6^	6.6	0.877	0.877	0.877	0.899	0.842	0.136
YOLO11-GCR	2.62 × 10^6^	6.2	0.919	0.922	0.921	0.913	0.895	0.147

**Table 4:** Performance of YOLO11-GCR on validation set vs. test set. The validation set consists of 30% of the 2024 data, while the test set is an independent collection from the 2025 survey.

Metric	Validation Set	Test Set
Precision	0.921	0.919
Recall	0.940	0.922
F1-score	0.930	0.921
mAP@0.5	0.977	0.913
mAP@0.5–0.95	0.843	0.895

## Data Availability

The raw data supporting the conclusions of this article will be made available by the authors on request.
